# Retinoic acid accelerates downregulation of the *Xist *repressor, Oct4, and increases the likelihood of *Xist *activation when *Tsix *is deficient

**DOI:** 10.1186/1471-213X-10-90

**Published:** 2010-08-20

**Authors:** Janice Y Ahn, Jeannie T Lee

**Affiliations:** 1 Howard Hughes Medical Institute Department of Molecular Biology, Massachusetts General Hospital Department of Genetics, Harvard Medical School Boston, MA 02114 USA; 2 Department of Molecular and Cellular Biology, Harvard University Cambridge, MA 02138 USA

## Abstract

**Background:**

Imbalances in X-linked gene dosage between the sexes are resolved by transcriptionally silencing one of two X-chromosomes in female cells of the early mammalian embryo. X-inactivation is triggered by expression of the non-coding *Xist *gene. In turn, *Xist *is dually regulated by the antisense Tsix RNA and by the Oct4 pluripotency factor. Although there is general agreement that *Tsix *is an inhibitor of *Xist*, some laboratories have observed ectopic *Xist *induction in differentiating male ES cells when *Tsix *is mutated, whereas we have not observed significant changes in *Xist*. These observational differences have led to fundamentally diverse models of X-chromosome counting. Here, we investigate if different methods of cell differentiation and use of all *-trans *retinoic acid (RA) could be causative factors and how they might impact *Xist *expression.

**Results:**

We compared suspension and cell-adhesion cultures in the presence or absence of RA and find that RA significantly impacts *Xist *expression in *Tsix*-mutant male cells. Whereas the standard embryoid body method infrequently leads to ectopic Xist expression, adding RA generates a significant number of Xist-positive male cells. However, while normal Xist clouds in wild-type female cells are robust and well-circumscribed, those found in the RA-treated mutant males are loosely dispersed. Furthermore, ectopic Xist expression does not generally lead to complete gene silencing. We attribute the effect of RA on *Xist *to RA's repressive influence on Oct4, a pluripotency factor recently shown to regulate *Tsix *and *Xist*. RA-treated ES cells exhibit accelerated decreases in Oct4 RNA levels and also display accelerated loss of binding to *Xist *intron 1. When *Tsix *is deficient, the faster kinetics of Oct4 loss tip the equilibrium towards *Xist *expression. However, the aberrant Xist clusters are unlikely to explain elevated cell death, as X-linked silencing does not necessarily correlate with the qualitatively aberrant Xist clusters.

**Conclusions:**

We conclude that RA treatment leads to premature downregulation of Oct4 and partial derepression of *Xist *irrespective of X-chromosome counting. RA-induced Xist clusters in male cells do not result in global or stable silencing, and excess cell death is not observed. These data and RA's known pleiotropic effects on ES transcription networks suggest that RA differentation bypasses normal X-inactivation controls and should be used judiciously. We propose that the likelihood of *Xist *expression is determined by a balance of multiple *Xist *activators and repressors, and that levels of Oct4 and Tsix are crucial toward achieving this balance.

## Background

Sex dosage compensation ensures equal X-linked gene expression between XX and XY individuals. In mammals, this balance is achieved by transcriptionally silencing an entire X-chromosome in females through a process called X-chromosome inactivation (XCI) [1]. XCI is accomplished independently in each cell primarily by the *Xist*/*Tsix *pair of sense/antisense non-coding RNAs located within the X-inactivation center (*Xic*) [2-5]. *Xist *upregulation and *cis*-coating of an X-chromosome represent important steps in the X-inactivation process [6,7], and are followed by recruitment of the PRC2 complex [8-10] and other silencing factors to initiate chromosome-wide inactivation and compaction into heterochromatin. Because X-inactivation occurs very early in embryonic development, cell culture models have been developed to facilitate analysis. Female mouse embryonic stem (ES) cells can undergo random X-inactivation when differentiated *in vitro *[2] and have therefore served as a powerful system with which to study this phenomenon. *Xist *expression remains low on both Xs in undifferentiated ES cells, but upon differentiation, *Xist *becomes upregulated only on the future inactive X [11,12]. *Xist *thus serves as the trigger for the silencing step during the X-inactivation program.

*Xist*'s central nature to XCI has led to intensive investigation of how this gene is regulated. One established regulator is *Tsix*, the antisense repressor of *Xist *[13,14]. Tsix RNA is expressed from both Xs in undifferentiated female cells, but its expression becomes monoallelic during the process of cell differentiation and XCI. The chromosome that transiently retains Tsix expression becomes the future active X (Xa), while the chromosome that extinguishes Tsix expression first becomes the future inactive X (Xi). *Tsix *has been proposed to regulate X-chromosome pairing, counting, and the mutually exclusive choice of Xa and Xi [15-17]. Indeed, various knockouts of *Tsix *(and its upstream enhancer *Xite *[18]) have led to nonrandom XCI as well as effects on counting and pairing [13-16].

Although *Tsix*'s repressive role in female cells seems clear, there has been debate over *Tsix*'s role in male cells. The original 3.7 kb deletion encompassing the major *Tsix *promoter in male ES cells (*Tsix*^ΔCpG^/Y [13]) did not cause a significant degree of ectopic *Xist *upregulation upon differentiation (<1%). *Tsix*^ΔCpG^/Y ES cells were phenotypically normal and capable of generating chimeric mice with germline transmission, and male offspring were born at expected frequencies [13]. This finding led to the conclusion that, whereas *Tsix *represses *Xist*, an additional factor - missing in male ES cells and present only in female cells - is required to induce *Xist *expression in cells with supernumerary X-chromosomes. It was therefore proposed that X-chromosome counting involves two factors: a 'blocking factor' that blocks *Xist *expression through *Tsix *on the Xa, and a 'competence factor' that induces *Xist *expression on Xi [13].

**Figure 1:**
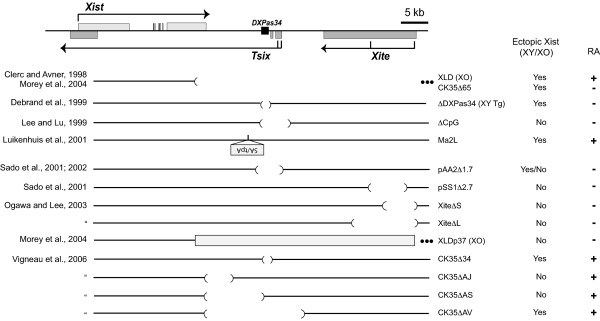
**Summary of Xist phenotypes in *Tsix*-mutant male ES cell lines**. All mutant ES cell lines are XY diploid male (except where noted) and listed with the names used in the original references. XY Tg, male transgenic line; shaded rectangle represents the 37-kb region re-inserted into a deleted locus; •••, extends beyond the *Xic *diagram depicted; RA, retinoic acid. SA/tpA, please refer to cited references for details. The 'yes/no' designation for pAA2Δ1.7 refers to the very low number of Xist-positive cells and the fact that there is no excessive cell death or inviability reported in these cells or mice. Differentiation in the presence (+) or absence (-) of retinoic acid is indicated.

Significantly, however, other *Tsix *mutations show variable phenotypes in male cells (Fig. 1). Sado et al. also showed that *Tsix*+/- female mice can yield viable male offspring carrying the mutation, but 8-21% of cells isolated from E7.5 male embryos showed ectopic Xist expression, an observation that could be explained by either ectopic Xist expression in the embryo proper or by presence of extraembryonic tissue (which is subject to imprinted XCI and therefore be severely affected by the maternal *Tsix *deletion) [14]. Another *Tsix-*specific mutation showed upregulation of Xist RNA in as many as 38% of differentiating male ES cells in culture [19,20]. A 65 kb deletion of the region 3' to *Xist *(including *Tsix*) also resulted in significant induction of Xist RNA clouds when XO (XLD [21]) or XY (CK35Δ65 [22]) cells underwent differentiation. Furthermore, smaller mutations including only *Tsix *elements were also found to have ectopic Xist cloud formation in a subset of differentiating male cells (CK35Δ34 and CK35ΔAV [19]). These observations have led to the alternative hypothesis that *Xist *is activated by default when *Tsix *is mutated, obviating the need for a competence factor involved in female-specific expression of *Xist*. Differences with regard to Xist expression in *Tsix*-deficient male cells (hereafter referred to as X^Δ^/Y ES cells) have therefore led to fundamentally different models for X-chromosome counting [3,13,15,22,23].

Here, we seek to understand why analyses of male cells in different laboratories should lead to differing results. Although the nature of the mutations could lead to disparate results, another potential difference among the studies might be the method of cell differentiation used to induce XCI. Whereas we differentiate ES cells by the embryoid body method (EB) in the absence of LIF, many laboratories also add retinoic acid (RA) to the differentiation medium. Although some studies have used both the EB and EB+RA methods and found ectopic Xist RNA in both cases [22,24], it remains formally possible that the combination of induction methods, culture conditions, and nature of the mutations could affect the Xist phenotype.

The idea that RA affects Xist expression finds support in two recent studies that demonstrated a role for Oct4 in repressing *Xist *by direct and indirect means [25,26]. One study showed that Oct4 positively regulates *Tsix *and that downregulation of Oct4 during cell differentiation triggers X-chromosome pairing, leads to monoallelic repression of *Tsix*, and consequently enables *Xist *upregulation on one chromosome [26]. The other study showed that Oct4 also associates with intron 1 of the *Xist *gene and proposed that Oct4 directly represses *Xist *[25]. Combined, these studies suggest that *Xist *is linked to cell differentiation through Oct4's effects on *Tsix *and *Xist*. Because it is known that Oct4 is rapidly downregulated in the presence of retinoic acid [27-30] (by binding the regulatory nuclear hormone receptors, RAR and RXR [31]), we consider the possibility that RA treatment to induce cell differentiation may have unanticipated effects on XCI and thereby confound XCI analysis. Here, we set out to test the idea that RA may affect *Xist *levels through Oct4 by studying one well-characterized *Tsix *mutation, *Tsix*^ΔCpG^, in male ES cells. Our results indicate that differentiation methods indeed influence *Xist *expression in these ES cells.

## Results

### Ectopic *Xist *upregulation in *Tsix*^ΔCpG ^male ES cells differentiated in the presence of retinoic acid

We differentiated wild-type and *Tsix*^ΔCpG ^male and female ES cells using variations of four published *in vitro *differentiation techniques: (1) embryoid body formation (EB method), whereby ES cell clusters are cultured in suspension for 4 days, in the absence of LIF, and then plated onto a solid matrix to form EB outgrowths; (2) EB method in the presence of 100 nM of all-*trans *retinoic acid (EB+RA method); (3) adherent cultures whereby ES cells are plated at low-density on gelatinized tissue culture plates without feeders and LIF (TC method); and (4) TC method in the presence of 100 nM all-*trans *retinoic acid (TC+RA method).

**Figure 2:**
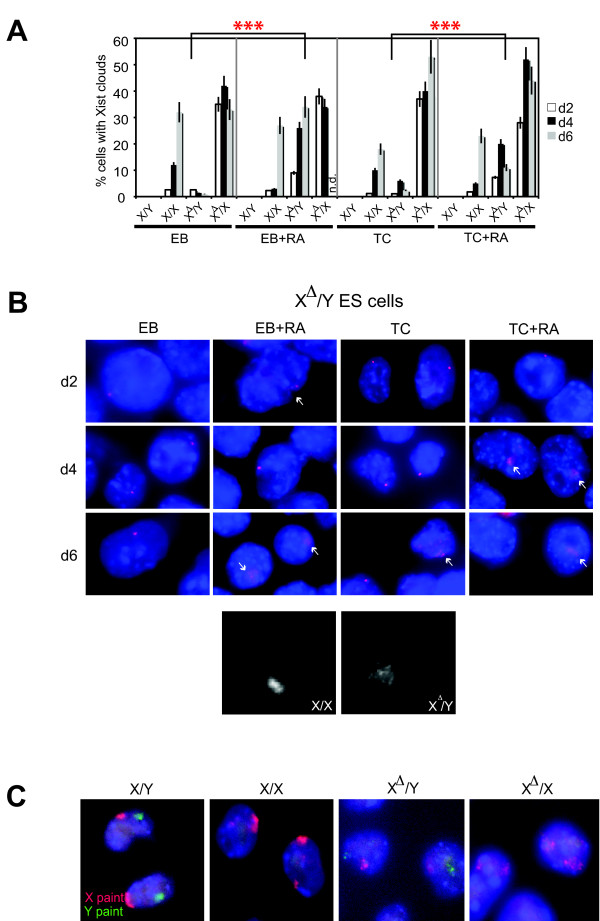
**Ectopic *Xist *RNA clouds observed in *Tsix*-mutant male ES cells**. **A**. Percentage of cells containing *Xist *RNA clouds. Note the high percentage of *Xist*-positive cells in X^Δ^/Y cells when differentiated in the presence of RA. Days of differentiation are listed as day 2 (d2), day 4 (d4), and day 6 (d6). Day 6 X^Δ^/X EB+RA, not determined (n.d.). X/Y, wild-type male ES line; X/X, wild-type female ES line; X^Δ^/Y, Tsix^ΔCpG^/Y male ES line; X^Δ^/X, Tsix^ΔCpG/+ ^female ES line; *n *> 200 for each timepoint and cell line examined. Error bars represent the standard deviation from three independent biological replicates. *P *was calculated by paired, two-tailed Student's *t*-tests. Differences in the number of Xist^+ ^cells for RA-free versus RA-induced X^Δ^/Y cells were statistically significant on all differentiation days (each red asterisk denotes *P *< 0.05 for day 2, day 4, and day 6 comparisons). EB, EB+RA, TC, and TC+RA conditions are described in the Materials and Methods. **B**. Xist RNA FISH showing ectopic and diffuse Xist cluster formation (arrows) in differentiating Tsix^ΔCpG ^male cells. Arrows denote Xist RNA clusters. The RNA FISH probe is double-stranded. Pinpoint signals primarily detect mildly elevated Xist expression in mutant cells caused by the *Tsix *deficiency, though ~10% residual Tsix expression from an upstream promoter may also contribute [13]. Below is a side-by-side comparison at day 6 (in grayscale) of a normal compact Xist RNA cloud (X/X, left) next to the ectopic dispersed Xist cluster observed in X^Δ^/Y cells (right). **C**. X- and Y-chromosome paint confirm that all ES cell lines carry the appropriate number of sex chromosomes. Red, X-chromosome; green, Y-chromosome.

By *Xist *RNA FISH (fluorescence *in situ *hybridization), we detected a slight increase in the percentage of cells with Xist clouds using the embryoid body (EB) versus the low-density cell adhesion (TC) method (Fig. 2A, 32% in X/X EB compared to 18% in X/X TC at day 6). Consistent with previous analysis [13], Xist clouds were rare in X^Δ^/Y cells in the absence of RA (<2-5%, *n*>200 cells) and were never observed in wild-type male cells under any differentiation condition and time point examined.

However, while no significant differences were seen for most cell lines between EB and EB+RA or between TC and TC+RA methods, we did detect differences for X^Δ^/Y cells (Fig. 2A,B). The differences were significant for all differentiation days (bracketed pairwise comparisons, *P *< 0.05). Ectopic Xist RNA clusters could be seen in 10-30% of X^Δ^/Y cells (*n *> 200) when induced to differentiate with RA. The ectopic Xist clouds were almost always more dispersed than wild-type Xist clouds, which are generally intense, compact, and well-defined (Fig. 2B, lower panels). This observation suggested a problem with either full *Xist *induction or transcript localization in mutant cells when differentiated with RA. We refer to these abnormal clouds as 'dispersed clusters' to emphasize these qualitative differences from wild-type Xist RNA clouds. X- and Y-chromosome DNA FISH confirmed that all ES cell lines used in this study exhibited the correct XX or XY constitution (Fig. 2C).

We conclude that the method of cell differentiation can impact *Xist *expression in X^Δ^/Y ES cells. Specifically, differentiation in the presence of RA leads to the appearance of ectopic Xist clusters only when *Tsix *is deleted and, when they do appear, the RNA is loosely clustered.

### RA induction accelerates loss of Oct4 binding to *Xist*

**Figure 3:**
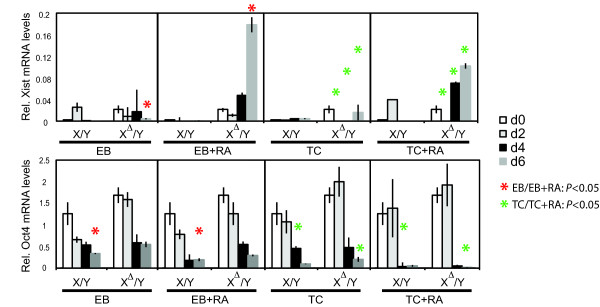
**RA-induced differentiation causes sharper decreases in Oct4 mRNA levels**. Xist and Oct4 RNA levels in wild-type and *Tsix*-mutant male ES cells under various differentiation conditions. Error bars represent one standard deviation from the mean. Days of differentiation are listed as day 0 (d0), day 2 (d2), day 4 (d4), and day 6 (d6). Pairwise comparisons were made between corresponding samples at each differentiation time point for EB vs. EB+RA (red asterisks) and TC vs. TC+RA (green asterisks). * *P *< 0.05. Statistical significance was calculated using paired, two-tailed Student's *t*-tests.

Recent studies suggest that *Xist *is negatively regulated not only by *Tsix *but also by Oct4 [25,26]. Because RA has been shown to negatively regulate the murine *Oct4 *promoter during ES cell differentiation [27-30], we asked if increased *Xist *expression might be due to accelerated Oct4 loss; i.e., in the absence of the second regulatory arm (*Tsix*), RA-treated cells would ectopically express *Xist*. By real-time RT-PCR, we compared relative *Xist *and *Oct4 *expression levels in differentiating male ES cells using the four described differentiation methods. In wild-type male cells, *Xist *was rapidly downregulated upon cell differentiation in a manner that correlated with downregulation of Oct4. In X^Δ^/Y cells differentiated by the EB method, Xist RNA was mildly upregulated upon cell differentiation when Oct4 levels dropped (Fig. 3), but this modest upregulation of Xist did not result in spreading of Xist RNA along the X, consistent with prior findings [13]. Thus, *Tsix *represses *Xist *even in male ES cells, but its absence only modestly increases Xist levels [13,14].

Differentiation with RA revealed two differences. First, while wild-type male cells appropriately repressed *Xist *expression, X^Δ^/Y ES cells displayed a significant rise in overall Xist RNA levels using the RA method (Fig. 3). This is consistent with the observation that, when differentiated via the EB method, <2-5% of X^Δ^/Y cells showed Xist clusters, but this percentage increased to 10-30% in the presence of RA (Fig. 2A). Similar results were obtained for both EB+RA and TC+RA conditions. At no time of differentiation up to day 6, however, did Xist levels increase by more than 2- to 10-fold in mutant male cells. Thus, Xist upregulation is considerably blunted in RA-induced *Tsix*-deficient male cells when compared to the 30- to 100-fold induction that typically accompanies XCI in normal female cells (data not shown) [13,32-34]. These results showed that RA differentiation led to a higher likelihood of Xist induction in *Tsix*-deficient male ES cells.

Second, Oct4 downregulation generally occurred more quickly in both wild-type and mutant male ES cells when compared to the same cells grown in the absence of RA (Fig. 3; see pairwise *t-*tests done for each cell line comparing between EB/EB+RA (red asterisks) and TC/TC+RA (green asterisks)). While Oct4 RNA levels were similar in wild-type and mutant ES cells in the undifferentiated state, differentiation revealed a significant difference in Oct4 mRNA levels at days 4 and/or 6 dependent on whether RA was added to the culture or not. RA accelerated the rate of Oct4 decrease regardless of whether the ES cells were grown as EBs (compare EB versus EB+RA) or as adherent cells (compare TC versus TC+RA). The greatest difference was evident when adherent cells were used. Although Oct4 mRNA differences are small, cells at this early stage of differentiation may be more sensitive to subtle changes in levels since a specific range of Oct4 concentration is required to maintain ES cells as pluripotent stem cells [35]. We note that the cell-adhesion method of differentiation has also been used and shown to form ectopic Xist RNA in *Tsix*-deficient male cells [19,22].

**Figure 4:**
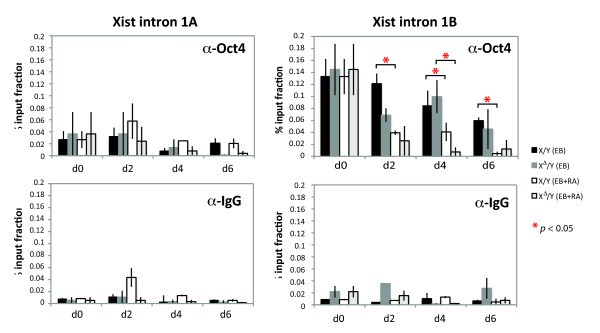
**Oct4-binding to *Xist *intron 1 decreases faster when differentiated in the presence of RA**. Quantitative ChIP analyses of Oct4-binding to *Xist *intron 1 show faster reductions in Oct4-binding kinetics when wild-type and *Tsix*-deficient male cells were differentiated by EB+RA versus EB methods. Note that, for each experiment, we started with a single d0 (undifferentiated) culture for both X/Y and X^Δ^/Y, and then split them on subsequent days for analysis. Thus, d0 samples were the same for all EB series and for all EB+RA series, and their values were duplicated in the graphs for easier comparisons with d2, d4, d6 samples in the same series. Means ± s.e.m from two independent biological replicates are shown. Pairwise comparisons were made between corresponding samples (EB vs. EB+RA) normalized to the control IgG ChIP (background) for each time point. Statistical significance of each result was calculated using paired, two-tailed Student's *t*-tests. * *p *< 0.05.

To determine whether the accelerated overall decrease in Oct4 levels has a direct impact on *Xist *expression, we asked if the kinetics of Oct4 loss at the *Xist *locus were altered in RA-containing versus RA-free cultures. Oct4 was previously shown to bind *Xist *intron 1 in ES cells [25]. It was proposed that Oct4 binding represses *Xist *in undifferentiated cells and that the loss of Oct4 binding during cell differentiation enables *Xist *upregulation. We therefore investigated whether cell differentiation triggered by RA might lead to accelerated loss of Oct4 binding to *Xist *intron 1 and increased *Xist *expression. By performing chromatin immunoprecipitations (ChIPs) using α-Oct4 antibodies on wild-type and *Tsix*-deficient male cells differentiated in parallel, we found that indeed Oct4-binding to *Xist *intron 1B was lost significantly more quickly when cells were differentiated in the presence of RA (Fig. 4; *P *< 0.05, see bracketed pairwise comparisons). We found that indeed Oct4-binding to *Xist *intron 1B was lost significantly more quickly when cells were differentiated in the presence of RA (Fig. 4; *P *< 0.05, see bracketed pairwise comparisons). Differences were evident as early as day 2. The difference was most evident for WT EB with versus without RA treatment; however, we note that the difference between mutant EB plus versus minus RA treatment might have been masked by somewhat lower Oct4 binding by d2. As a negative control, we looked at a region within intron 1 of *Xist *(intron 1A, located 0.6 kb away from intron 1B), which is not known to bind Oct4 [26], and found no statistically significant differences between EB and EB+RA methods. Thus, RA treatment leads to a more rapid loss of Oct4 binding to *Xist *intron 1 than is typically seen when ES cells are differentiated without RA.

Taken together, these data indicated that RA treatment has little effect on Xist RNA upregulation when *Tsix *is present, but increases the likelihood with which Xist will be induced when *Tsix *is deficient. We propose that RA's effect in the absence of *Tsix *is related to the rapid downregulation of Oct4 by RA, which in turn leads to accelerated loss of Oct4 binding to *Xist *intron 1. We suggest that, when these events occur in a *Tsix*-deficient background, *Xist *is more likely to become derepressed. Overall Oct4 levels are also downregulated in wildtype cells when treated with RA, but the persistence of *Tsix *would antagonize the derepressive forces. These findings are consistent with the idea that Oct4 and *Tsix *act in parallel to regulate *Xist *expression [25,26].

### X-chromosome silencing in mutant cells with dispersed Xist clusters

**Figure 5:**
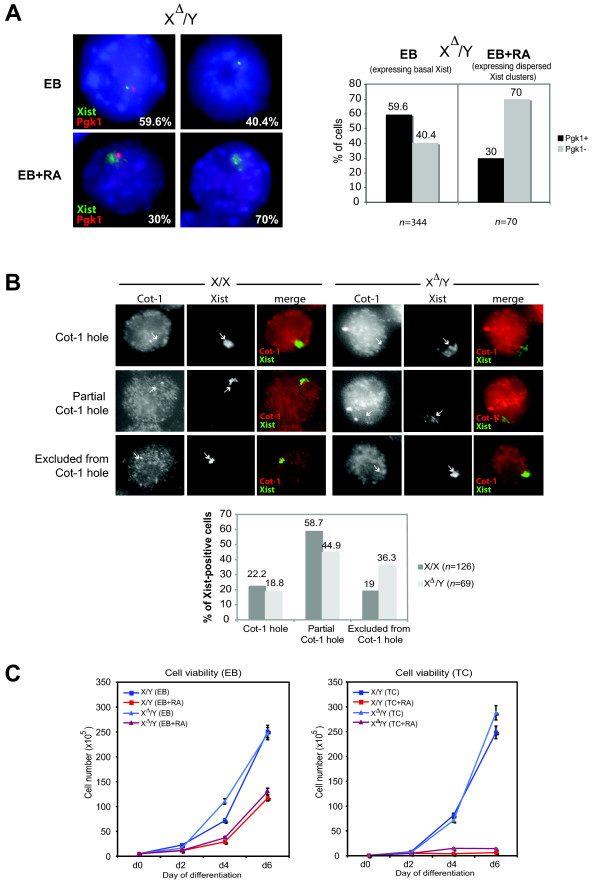
**RA-containing cultures lead to silencing of the X-linked gene *Pgk1 *and exhibit slower overall growth than cultures without RA**. **A**. Xist/Pgk1 RNA FISH showing the percentage of *Pgk1 *expression in X^Δ^/Y cells differentiated under the EB or EB+RA condition at day 6. EB+RA percentages represent only those cells positive for dispersed Xist RNA clusters. EB percentages represent all cells expressing basal Xist levels. **B**. Xist/Cot-1 RNA FISH of X/X (EB) and X^Δ^/Y (EB+RA) with Xist RNA clouds at day 6. Percentages represent Xist+ cells that were scored to be located within a Cot-1 RNA hole, partially within a Cot-1 hole, or excluded from a Cot-1 hole, as indicated. Note the higher percentage of Xist clouds excluded from a Cot-1 hole in X^Δ^/Y cells when compared to X/X cells. Arrows point to Xist clouds/clusters and their corresponding Cot-1 regions. **C**. Growth curves for wild-type and *Tsix*-mutant male cells differentiated under EB/EB+RA **(left) **or TC/TC+RA **(right) **conditions. Cultures containing RA (denoted in shades of red) had <50% of cells than those cultures grown in the absence of RA (denoted in shades of blue). No differences were seen between X/Y and X^Δ^/Y grown under similar conditions. Error bars represent one standard deviation from the mean; days of differentiation are listed as day 0 (d0), day 2 (d2), day 4 (d4), and day 6 (d6).

We next addressed whether the 2- to 10-fold increases in Xist levels and formation of dispersed Xist RNA clusters led to X-chromosome silencing in RA-treated *Tsix*-mutant cells. First, to correlate Xist expression with gene silencing on a cell-by-cell basis, we carried out RNA FISH using probes to simultaneously detect Xist and nascent transcription from the X-linked *Pgk1 *locus. In wild-type X/X cells that have undergone XCI, nascent Pgk1 RNA signals are generally not observed on the Xi, whereas they can be observed in ~60% on the Xa at a given time point [13] (Fig. 5A and data not shown). We reasoned that, if ectopic Xist clusters led to gene silencing in X^Δ^/Y cells, Pgk1 RNA would not be detectable on the Xist-coated X^Δ^. On the other hand, if the aberrant Xist clusters could not silence genes *in cis*, 60% of X^Δ ^would continue to express Pgk1. When differentiated using the RA method, 30% (21/70) of X^Δ^/Y cells with Xist accumulation showed nascent Pgk1 signals (Fig. 5A), suggesting that complete silencing did not always follow Xist accumulation at this time point, though a fraction of EB+RA cells appeared to undergo inactivation. By contrast, when differentiated using the EB method, X^Δ^/Y cells did not express Xist, and Pgk1 expression was observed at the expected frequency of ~60% (Fig. 5A). Thus, although *Xist *was partially derepressed by RA treatment of X^Δ^/Y cells, silencing of genes *in cis *did not proceed to completion.

With respect to XCI, the X can be divided into genic versus repetitive element fractions [36,37]. To assess whether repetitive element silencing proceeded to completion, we carried out RNA FISH using probes from the Cot-1 fraction [38], the genomic fraction containing the most highly repetitive elements. In cells where XCI is firmly established, the inactive X lies either completely or partially within a "Cot-1 hole" (i.e., the inactive X is depleted for repetitive element expression) [36-39]. In wild-type EB cells on day 6 of differentiation, 22.2% of Xist clouds lay entirely within a Cot-1 hole and 58.7% were partially within a Cot-1 hole; only 19% did not appear to overlap with a Cot-1 hole at all (Fig. 5B). By contrast, in RA-differentiated X^Δ^/Y EB, 18.8% of aberrant Xist RNA clusters were located within a Cot-1 hole and 44.9% partially within a Cot-1 hole, whereas 36.3% did not overlap with a Cot-1 hole at all (Fig. 5B). Because the ectopic Xist RNA clusters in X^Δ^/Y cells were very diffuse, Xist RNA could often be found in Cot-1^+ ^regions (and were scored as 'partially within a Cot-1 hole'). Interestingly though, Xist RNA clusters scored as 'entirely within a Cot-1 hole' appeared more compact, suggesting that perhaps only those cells were able to sufficiently silence their X-chromosomes. Taken together, these results demonstrated that a significant fraction of RA-treated mutant male cells had not completed repeat silencing, suggesting either a delay or a failure to do so. Thus, both genic and repeat silencing were at least partially compromised in X^Δ^/Y cells with ectopic and diffuse Xist expression induced by RA-mediated cell differentiation.

Next we reasoned that, if Xist induction by RA led to XCI in part, differentiating X^Δ^/Y cells would show cell death in excess of wild-type cells. However, an equivalent level of cell death was observed for mutant and wild-type cells, suggesting that the X^Δ ^chromosome could not have been silenced to a significant extent (Fig. 5C). Notably, regardless of the *Tsix *genotype, the magnitude of cell death was consistently greater and the population doubling time was substantially slower when cells were differentiated using the RA method (Fig. 5C). Comparison of EB versus EB+RA conditions showed that <50% of cells - either wild-type or mutant - survived RA treatment during the first 6 days. Comparison of TC versus TC+RA showed that addition of RA caused a >95% drop in cell viability over the same period. These results demonstrate that the *Tsix *mutation does not have a major effect on the viability of male cells and that RA alone - irrespective of the *Tsix *deficiency and RA's partial effect on Xist induction - has major effects on cell viability that are unrelated to the XCI pathway. This effect of RA raises the possibility of selection artifacts when mutations are analyzed in the presence of RA.

## Discussion

Here, we have shown that differentiation in the presence of RA results in partial upregulation of Xist RNA when *Tsix *is deleted in male ES cells. We believe that this results from accelerated loss of Oct4 and that the depletion of both *Tsix *and Oct4 (both repressors of *Xist*) in differentiating ES cells leads to ectopic Xist expression. However, the ectopic Xist clusters appear more dispersed than those typically seen in wild-type female cells, and the degree of Xist activation is an order of magnitude lower. Consistent with these aberrant qualities, the dispersed Xist clusters do not globally or stably silence the male X-chromosome. Thus, RA-induced Xist expression in a *Tsix*-deficient background does not lead to proper and complete XCI. We also observed that RA treatment uniformly causes massive cell death (>50%) regardless of the *Tsix *genotype. Nonetheless, the fraction of *Tsix*-deficient male cells that do initiate silencing may be selected against, thus enriching for cells that have not properly initiated XCI. In view of our findings, we suggest that RA differentiation of XCI mutants should be analyzed carefully because RA treatment could either create selection artifacts or indirectly affect XCI through RA's established pleiotropic effects.

It has long been known that RA regulates expression of the POU-domain transcription factor Oct4 (also referred to as *Oct3/4 *and *Pou5f1*), which is restricted to pluripotent stem cells and is downregulated when induced to differentiate by treatment with RA [27-30]. RA and other vitamin A derivatives have pervasive effects on developmental processes--including vertebrate embryogenesis, growth, differentiation, and homeostasis--primarily through binding to the RAR and RXR nuclear hormone receptors [31]. RAR isoforms mediate transcriptional activation through binding of either 9-*cis *or all-*trans *RA [40,41], while RXRs (α, β, γ) work specifically through 9-*cis*-RA [31,42-44]. These receptors form homo- or heterodimeric complexes and bind RA-responsive elements in target genes to transcriptionally mediate expression [45-49]. *Oct4 *is regulated at the level of transcription [35] through binding of RARs [50], chick ovalbumin upstream promoter transcription factors (COUP-TFs) [51], germ cell nuclear factor (GCNF), as well as other proteins [52], to upstream enhancer regions (designated DE and PE for distal and proximal enhancers, respectively) [50] and an Sp1 site [53] located within the *Oct4 *promoter. Upon RA-induced differentiation, these specific factors bind to and downregulate *Oct4 *in ES and EC cells [50,51,54]. It is possible that these genome-wide and pleiotropic effects of RA would have indirect effects on XCI, which might thereby explain differences in the *Xist *phenotype observed in our *Tsix-*deficient background from those of others.

**Figure 6:**
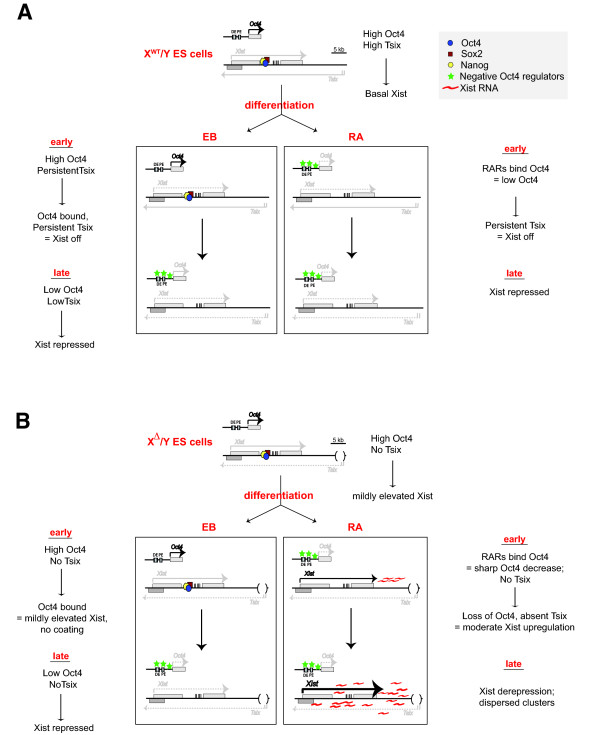
**Model of RA-induced *Xist *expression in *Tsix*-mutant male cells**. **A**. Undifferentiated wild-type X/Y cells maintain high levels of Oct4, Sox2, and Nanog, which bind to *Xist *intron 1. This synergistic binding -- in addition to high Tsix RNA expression -- effectively suppresses *Xist*. Under the EB differentiation method **(left)**, *Oct4 *expression remains high early during differentiation, allowing continued binding of Oct4, Sox2, and Nanog to keep *Xist *off. Although Oct4 levels and binding decrease during late differentiation, *Xist *continues to remain repressed as the *Xist*-dependent phase of XCI has passed. Under the RA differentiation method **(right)**, RA binds to its receptor to form RAR complexes, which then bind to and negatively regulate *Oct4*. However, persistent *Tsix *expression maintains stable *Xist *repression. **B**. In undifferentiated X^Δ^/Y cells, high levels of Oct4, Sox2, and Nanog are sufficient to repress *Xist *in the absence of *Tsix*. Under the EB differentiation method **(left)**, *Oct4 *expression remains high early during differentiation, allowing continued binding of Oct4, Sox2, and Nanog to maintain low *Xist *expression even in the absence of *Tsix *transcription. Although Oct4 mRNA levels and binding to *Xist *intron 1 decrease during late differentiation, *Xist *is stably repressed as the *Xist*-dependent phase has passed. However, in the RA differentiated state **(right)**, RAR complexes bind to *Oct4*, causing premature Oct4 downregulation; loss of Oct4 binding to *Xist *intron 1 and absence of *Tsix *lead to moderate *Xist *upregulation during the early (*Xist*-dependent) stages of differentiation. Due to *Xist *derepression during this early stage and lack of antisense *Tsix *transcription, Xist RNA starts to accumulate and diffusely coats the chromosome *in cis*, leading to formation of visible Xist RNA clusters and incomplete silencing. DE and PE refer to distal and proximal *Oct4 *enhancers, respectively.

Xist differences in EB- versus RA-differentiated cultures can be summarized by the model shown in Figure 6. Our model supposes that *Xist *is independently repressed by Oct4 and *Tsix*. In undifferentiated wild-type male ES cells (Fig. 6A), high levels of Oct4--along with Nanog and Sox2--bind within intron 1 of the *Xist *gene [25,26] and keep *Xist *expression at a "basal" level (<10 copies per cell) [8,55,56]. Oct4 also binds to *Tsix*/*Xite *to transactivate antisense transcription (not shown), which in turn also represses *Xist *[25,26]. During differentiation into EBs, *Oct4 *expression decreases slowly and remains high enough during early differentiation to maintain *Xist *repression. At the same time, persistent *Tsix *expression stably switches off *Xist *("off" state) [33,55,57-59]. This early stage corresponds to the reversible *Xist*-dependent time window for XCI [60]. Later during differentiation, *Oct4 *is significantly downregulated, *Tsix *is extinguished, and *Xist *is safely and stably repressed, possibly by a transcriptional gene silencing (TGS)-like mechanism and by DNA methylation of the *Xist *promoter [59,61] (after the *Xist*-dependent time window [60]). Under conditions of RA-induced differentiation and accelerated loss of Oct4, the outcome would be similar, as *Tsix *ensures continued repression of *Xist*.

In X^Δ^/Y cells, the outcome would be different (Fig. 6B). Ordinarily, the rapid drop in Oct4 levels during RA-induced differentiation would have little impact on *Xist*, as an intact *Tsix *would suppress full *Xist *activation. However, deletion of *Tsix *would eliminate the second arm of *Xist *regulation, resulting in ectopic Xist expression. The increase in *Xist *expression would be modest, would not yield robust Xist clouds, and would not necessarily be followed by chromosome-wide inactivation, explaining why we did not observe a noticeable increase in cell death.

Although we prefer this model, we cannot exclude other potential explanations for the behavior of *Tsix*^Δ*CpG*^/Y cells in general. It is clear that the *Xist *promoter is not properly silenced in RA-differentiated *Tsix*-mutant male cells. Therefore, it is possible that a very small minority of *Tsix*^Δ*CpG*^/Y cells that initiate XCI during the early time window (when the Xi can be established) are selected against and are rarely observed. Because *Xist *is not properly silenced in the absence of *Tsix*, cells might continue to upregulate *Xist *at later stages and form improper and abnormal Xist RNA clusters, which would not be capable of silencing the X. These cells might then survive longer and be observed by our assay. Although this scenario is possible, we do not believe that it can explain the observations related to RA made in this study. Firstly, ectopic *Xist *expression is rarely observed without RA treatment. Secondly, in the presence of RA, Xist RNA clusters are actually observed during the early time window during the silencing-competent phase of XCI. Thus, we believe that the more likely explanation is the one offered in Figure 6.

Interestingly, the percentage of RA-differentiated cells with ectopic *Xist *upregulation in a *Tsix*-mutant background is similar among other studies and our own (~10% at day 2) [19,20,22]. Our results are also consistent with those reported by Navarro et al. for an *Oct4*-null male ES line. Although these authors reported that *Xist *upregulation was not seen in *Oct4*-null male ES cells when differentiated by retinoic acid alone [25], this line contained a tetracycline-repressible *Oct4 *transgene which lacked the endogenous *Oct4 *promoter and upstream enhancer regions (PE and DE). As the retinoic acid repressible elements [50,62] are located within this region, their *Oct4*-null male line would not be directly affected by RA treatment.

Our results suggest that the likelihood of Xist expression is determined by a balance of multiple *Xist *activators and repressors, consistent with other proposed models [13,15,23,63-65]. Deletion, mutation or downregulation of repressive factors would predispose that X-chromosome to inactivation, but would ordinarily not lead to ectopic inactivation in male cells when other repressors are present. Thus, deletion of the *Tsix *locus alone in male cells would prevent binding of XCI repressive factors to the single X-chromosome but not lead to ectopic inactivation. However, the absence of *Tsix *in combination with premature loss of Oct4-binding to *Xist *intron 1 due to RA-induced differentiation in Tsix^ΔCpG^/Y male cells would be sufficient to derepress *Xist *expression independently from this sensing mechanism.

This idea may explain why some investigators have observed the appearance of Xist clusters even when *Tsix*-deficient cells were differentiated using the EB method. In our hands, *Tsix*^ΔCpG^/Y ES cells rarely show Xist clusters (<5%). Sado et al. have reported that the *Tsix*^pAA2^^Δ1.7^/Y mutant shows slightly more Xist clusters during early differentiation by the EB method (~10%) [14,24], which may be explained by the severity of this *Tsix *mutation. The *Tsix*^pAA2^^Δ1.7 ^mutation is a severe truncation mutation with lethality being highly penetrant in the mouse [14,24], whereas the *Tsix*^ΔCpG ^mutation shows ~10% residual antisense expression and shows lower penetrance of lethality in mice [13,66]. The combination of a severe *Tsix*^pAA2^^Δ1.7 ^mutation with RA-accelerated Oct4 depletion could explain why *Xist *upregulation is more frequently seen than in the case for our *Tsix*^ΔCpG ^mutation. The 65 kb deletion of Avner and colleagues yields an even greater number of Xist^+ ^cells when differentiated into EBs [19,22]. Because the 65 kb deletion includes all of the 5' end of *Tsix *and *Xite *and deletes two of the three known Oct4-binding sites within the *Xic *[25,26], we propose that combining it with accelerated decreases in both RA-induced Oct4 transcription and binding would further increase the likelihood of *Xist *expression.

## Conclusions

We argue that the moderate elevation of Xist RNA levels in RA-treated X^Δ^/Y cells is not due to aberrant X-chromosome counting but to premature Oct4 downregulation and accelerated loss of Oct4-binding to *Xist *intron 1. RA also has general pleiotropic effects on transcription that could further affect the XCI process. Our observations suggest that cells lacking *Tsix *- when differentiated under more physiological conditions (such as that simulated by LIF withdrawal and formation of EBs) - remain capable of suppressing *Xist *and preserving viability. These ideas are consistent with the two-factor model, which proposes that a repressive "blocking" factor and an activating "competence" factor coordinately regulate the X-chromosome counting process [13,15]. By this model, Xist RNA is induced and XCI is initiated only when the blocking factor is not bound *in cis *and a competence factor (expressed only in X/X cells) is present. Both X/Y and X^Δ^/Y cells would lack the competence factor and be incapable of fully initiating XCI even if *Tsix *and the blocking factor were eliminated. Apart from the consequences shown here, RA's acceleration of Oct4 downregulation would also be expected to alter the timing of X-chromosome pairing, an event proposed to regulate X-chromosome counting, and thereby account for why different studies observe pairing on different differentiation days [16,17]. Relevantly, Oct4 has been shown to control the timing of X-chromosome pairing [26]. We suggest, therefore, that RA should be used judiciously in studies of X-chromosome counting and other events surrounding the initiation of XCI.

## Methods

### ES cell lines and culture

Wild-type male (J1), female (EL16.7), and *Tsix*-mutant (CG7 male and 3F1 female) ES cell lines and culture conditions have been previously described [13].

### ES cell differentiation

Each ES cell line was differentiated for six days using one of the following differentiation methods: (1) suspension cultures forming embryoid bodies (EB), (2) suspension cultures forming EBs + 100 nM of all-*trans *retinoic acid (EB+RA), (3) cells plated at low density on 0.2% gelatinized tissue culture plates (TC), and (4) cells plated at low density on 0.2% gelatinized tissue culture plates + 100 nM all-*trans *retinoic acid (TC+RA). LIF (leukemia inhibitory factor) was removed in all differentiation methods. EB and EB+RA cultures had a starting concentration of ~5 × 10^5 ^cells/60 cm^2^, while TC and TC+RA cultures had a starting concentration of ~1 × 10^5 ^cells/60 cm^2^. Culture medium was changed every two days, and 100 nM all-*trans *retinoic acid was added fresh where appropriate. All-*trans *retinoic acid (Sigma Cat # R2625) was diluted in DMSO as 10 mM stock solutions, stored in light-protected vials at -20°C, and diluted in culture media just prior to use. All experiments were performed three times. Viable cells were counted using a Cellometer Auto cell counter (Nexcelom Biosciences).

### RNA fluorescence *in situ *hybridization

ES cells were trypsinized into single cells, cytospun onto glass slides, fixed in 4% paraformaldehyde/1xPBS, and FISH was carried out as described [13]. *Xist*, *Pgk1*, and Cot-1 RNA were detected using double-stranded DNA probes labeled with Cy3-dUTP or FITC-dUTP either by nick-translation (Roche) or using the Prime-It Fluor Fluorescence Labeling Kit (Stratagene). Digital images were taken with a Nikon Eclipse 90i microscope (Nikon Instruments, Inc.) and processed using Volocity software (Improvision). In brief, *z*-sections were captured at 0.2 μm intervals, and 3-D images were projected onto a single 2-D plane. Cells were counted and scored for the presence or absence of an *Xist *cloud or cluster. X- and Y-chromosome paint was performed on undifferentiated cells as previously described [67] using StarFISH Cy3- or FITC-labeled probes from Cambio.

### Real-time RT-PCR

Total cellular RNA was isolated from TRIzol reagent (Invitrogen) by phenol/chloroform extraction and treated with TURBO DNase (Ambion). RNA (1 μg) was reverse transcribed into cDNA with random hexamers using the SuperScript III First-Strand Synthesis System (Invitrogen) following the manufacturer's instructions. Real-time PCR was performed on a Bio-Rad iCycler machine with SYBR-Green iQ Mix (Bio-Rad). After an initial denaturation at 95°C for 8:30 min, reactions were amplified for 40 cycles: 95°C for 30 s, 58°C for 30 s, 72°C for 30 s, followed by dissociation curve analyses at 55°C for 1 min and 55°C + 0.5°C every 20 s. *Xist *primers amplified exons 1-3 of the cDNA and were as follows: NS66, 5'-GCTGGTTCGTCTATCTTGTGGG-3' and NS33, 5'-CAGAGTAGCGAGGACTTGAAGAG-3' [34]. *Oct 4 *and *GAPDH *primers were as follows: Oct4F, 5'-GAAGCAGAAGAGGATCACCTTG-3'; Oct4R, 5'-TTCTTAAGGCTGAGCTGCAAG-3'; GAPDH1F, 5'-ATGAATACGGCTACAGCAACAGG-3'; GAPDH1R, 5'-CTCTTGCTCAGTGTCCTTGCTG-3'. All primers were added at a final concentration of 200 nM. The Ct (defined as the cycle number at which the fluorescence exceeds the threshold value) was determined for each reaction run in triplicate, and relative-fold differences were calculated using the 2^-^^ΔΔCt ^method normalized to GAPDH levels and female fibroblasts (for Xist) or undifferentiated ES cells (for Oct4) as a reference sample [68].

### Chromatin immunoprecipitations (ChIPs)

ChIP analyses were carried out using a modified protocol from Millipore, and the results were averaged for two independent biological replicates of wild-type and Tsix^ΔCpG ^male cells at day 0, day 2, day 4, and day 6 of differentiation; error bars denote standard errors of the means.

#### Chromatin isolation

Briefly, 1-2 × 10^7 ^cells were trypsinized into a single-cell suspension and crosslinked with formaldehyde to a final concentration of 1% at 37°C for 10 min. Crosslinking was quenched with glycine (125 mM final), and cells were pelleted at 640 *g *for 5 min and washed twice with 1× phosphate buffered saline containing protease inhibitors (Roche mini cOmplete protease inhibitor cocktail tablets, EDTA-free #11-836-170-001). Nuclei were isolated from fixed cells by washing once with Buffer A-NP-40 (5 mM PIPES, pH 8.0, 85 mM KCl, 0.5% NP-40), incubated on ice for 10 min, then washed with Buffer A (5 mM PIPES, pH 8.0, 85 mM KCl) and Lysis Buffer (10 mM Tris-HCl, pH 8.0, 10 mM NaCl, 3 mM MgCl_2_, 0.5% NP-40). Pelleted nuclei were resuspended in MNase buffer (10 mM Tris-HCl, pH 8.0, 10 mM NaCl, 3 mM MgCl_2_, 1 mM CaCl_2_, 4% NP-40) containing protease inhibitors and 1% SDS. Lysates were sonicated using the Bioruptor XL (Diagenode) [15 min total with 30 s on- and 30 s off-cycles]. Sonicated lysates were centrifuged at 16,100 *g *for 10 min, and the supernatants were stored at -80°C.

#### Immunoprecipitation

All IP steps were performed at 4°C. For each immunoprecipitation, 50 μl of Dynal Protein G-magnetic beads (Invitrogen # 100-04D) were first incubated with 5 μg of appropriate antibody for 2 hours with rotation, then with sonicated supernatants (~2.0 × 10^6 ^cells) overnight. IP samples were washed twice each with Low Salt TSE 150 (20 mM Tris-HCl, pH 8.0, 0.1% SDS, 1% Triton X-100, 2 mM EDTA, 150 mM NaCl), High Salt TSE 500 (20 mM Tris-HCl, pH 8.0, 0.1% SDS, 1% Triton X-100, 2 mM EDTA, 500 mM NaCl), LiCl Buffer (10 mM Tris-HCl, pH 8.0, 250 mM LiCl, 1% NP-40, 1% deoxycholate, 1 mM EDTA), and TE Buffer (10 mM Tris-HCl, pH 8.0, 1 mM EDTA). Protein/antibody complexes were eluted from the beads with freshly made Elution Buffer (50 mM Tris-HCl, pH 8.0, 1 mM EDTA, 1% SDS, 50 mM NaHCO_3_) incubated at 65°C for 10 min. Crosslinks were reversed by digestion with 80 μg proteinase K at 65°C for 4 hours, and DNA was recovered by phenol/chloroform extraction.

ChIP antibodies included goat polyclonal anti-Oct4 (Santa Cruz #sc8628) and normal rabbit IgG (Cell Signaling #2729). Quantitative PCR was performed using an iCycler iQ real-time PCR detection system (Bio-Rad) with primers specific to *Xist *intron 1 (site A: p63/p64, distanced 0.6 kb from site B: p65/p66). Primer sequences were as follows: p63, 5'-CTGAAGATGGTGATGGCGAGTTG-3'; p64, 5'-AAAGAGTTCCCCAAATTAGTGTCCTG-3'; p65, 5'-ATGTTTCCTTTTGAAGCAGTTACTTGTAC-3'; p66, 5-CATTGTCTGGCTCTCTAGGTGATAATAC-3' [26]. Pairwise comparisons were made between corresponding samples (EB vs. EB+RA) normalized to the control IgG ChIP (background) for each time point. Statistical significance of each result was calculated using a paired, two-tailed *t*-test. * *p *< 0.05

## Authors' contributions

JYA performed all of the experiments. JYA and JTL designed the experiments, analyzed the data, and wrote the paper. All authors read and approved the final manuscript.
